# Equivalence of electronic and paper administration of patient-reported outcome measures: a systematic review and meta-analysis of studies conducted between 2007 and 2013

**DOI:** 10.1186/s12955-015-0362-x

**Published:** 2015-10-07

**Authors:** Willie Muehlhausen, Helen Doll, Nuz Quadri, Bethany Fordham, Paul O’Donohoe, Nijda Dogar, Diane J. Wild

**Affiliations:** ICON Clinical Research, 6th Floor Seacourt Tower, West Way, Oxford, OX2 0JJ UK; CRF Health, Brook House - 3rd Floor, 229-243 Shepherds Bush Road, Hammersmith, London, W6 7AN UK

**Keywords:** Equivalence, Meta-analysis, Pen and paper, Web/computer platform, IVRS platform, Tablet/touchscreen platform, PDA/smartphone platform

## Abstract

**Objective:**

To conduct a systematic review and meta-analysis of the equivalence between electronic and paper administration of patient reported outcome measures (PROMs) in studies conducted subsequent to those included in Gwaltney et al’s 2008 review.

**Methods:**

A systematic literature review of PROM equivalence studies conducted between 2007 and 2013 identified 1,997 records from which 72 studies met pre-defined inclusion/exclusion criteria. PRO data from each study were extracted, in terms of both correlation coefficients (ICCs, Spearman and Pearson correlations, Kappa statistics) and mean differences (standardized by the standard deviation, SD, and the response scale range). Pooled estimates of correlation and mean difference were estimated. The modifying effects of mode of administration, year of publication, study design, time interval between administrations, mean age of participants and publication type were examined.

**Results:**

Four hundred thirty-five individual correlations were extracted, these correlations being highly variable (I2 = 93.8) but showing generally good equivalence, with ICCs ranging from 0.65 to 0.99 and the pooled correlation coefficient being 0.88 (95 % CI 0.87 to 0.88). Standardised mean differences for 307 studies were small and less variable (I2 = 33.5) with a pooled standardised mean difference of 0.037 (95 % CI 0.031 to 0.042). Average administration mode/platform-specific correlations from 56 studies (61 estimates) had a pooled estimate of 0.88 (95 % CI 0.86 to 0.90) and were still highly variable (I2 = 92.1). Similarly, average platform-specific ICCs from 39 studies (42 estimates) had a pooled estimate of 0.90 (95 % CI 0.88 to 0.92) with an I2 of 91.5. After excluding 20 studies with outlying correlation coefficients (≥3SD from the mean), the I2 was 54.4, with the equivalence still high, the overall pooled correlation coefficient being 0.88 (95 % CI 0.87 to 0.88). Agreement was found to be greater in more recent studies (*p* < 0.001), in randomized studies compared with non-randomised studies (*p* < 0.001), in studies with a shorter interval (<1 day) (*p* < 0.001), and in respondents of mean age 28 to 55 compared with those either younger or older (*p* < 0.001). In terms of mode/platform, paper vs Interactive Voice Response System (IVRS) comparisons had the lowest pooled agreement and paper vs tablet/touch screen the highest (*p* < 0.001).

**Conclusion:**

The present study supports the conclusion of Gwaltney’s previous meta-analysis showing that PROMs administered on paper are quantitatively comparable with measures administered on an electronic device. It also confirms the ISPOR Taskforce´s conclusion that quantitative equivalence studies are not required for migrations with minor change only. This finding should be reassuring to investigators, regulators and sponsors using questionnaires on electronic devicesafter migration using best practices. Although there is data indicating that migrations with moderate changes produce equivalent instrument versions, hence do not require quantitative equivalence studies, additional work is necessary to establish this. Furthermore, there is the need to standardize migration practices and reporting practices (i.e. include copies of tested instrument versions and screenshots) so that clear recommendations regarding equivalence testing can be made in the future.raising questions about the necessity of conducting equivalence testing moving forward.

## Introduction

The implementation of electronic data capture (EDC) in clinical trial settings has become more commonplace as the use of electronic devices in everyday life has become more widespread. Tablets and smart phones are used universally across many age groups [1, 2] and prior experience is not a prerequisite for their use [3]. Smart phone subscription is expected to reach 5.6 billion by 2019 [4]. The advantages of using EDC for the administration of patient-reported outcome measures (PROMs) rather than paper-and-pencil administration have been well documented; these include reduction in administrative burden, automatic implementation of skip patterns and scoring, avoidance of secondary data entry errors, time and date stamped data, and fewer items of missing data [5].

The FDA states in its Final PRO Guidance document [6] that the migration of validated paper instruments to electronic platforms should be supported with evidence: “additional validation to support the development of a modified PRO instrument” is required, including when “an instrument’s data collection mode is altered”, with specific reference to “paper-and-pencil self-administered PRO administered by computer or other electronic device (e.g., computer adaptive testing, interactive voice response systems, web-based questionnaire administration, computer)” (p.20-21).

There is, however, lack of clarity in the FDA guidance document on the type of evidence required to support PRO to ePRO migrations. As a consequence, the ISPOR ePRO Task Force, led by Stephen Coons, was established to address this issue [7]. This Task Force developed recommendations on how to demonstrate measurement equivalence between electronic and paper-based PROMs, where measurement equivalence refers to the comparability of the conceptual and psychometric properties of the data obtained via the two administration modes [7]. In this respect, the level of modification to the content and format of the paper PROM to produce an electronic version (and, increasingly, between various electronic modes) determines how comparable the two versions are and thus the evidential requirements to demonstrate equivalence between versions.

Coons et al. [7] categorised the magnitude of the modification into three levels, whereby the potential effect on the content, meaning, or interpretation of the measure’s items and/or scales is assessed. If a paper-and-pencil questionnaire is simply placed into a text screen format without significantly altering item content, recall period or response options, this is considered a minor modification. Minor levels of modification also include going from multiple items per page to one item per screen, for example on a handheld device. The level of evidence required to show equivalence for a minor modification is cognitive interviewing and usability testing.

Where a modification is considered to be moderate, Coons et al. [7] suggest that the modification may result in changes to the (perceived) meaning of the assessment items. Examples of moderate changes include splitting an item into multiple screens (e.g., having a question and its responses on different screens), using a scroll bar to view all item text or responses, and changing the order of item presentation. Where such modifications are made, the level of evidence required would involve conducting quantitative equivalence testing, which evaluates the comparability between PROM scores from the electronic mode of administration and the original mode. The intent is to ensure scores do not vary significantly between modes, barring measurement error. Usability testing is also recommended, to ensure prospective participants experience no issues with the usability of the device. The most common moderate change is from a text based to an interactive voice response system (IVRS). This is considered to be a moderate change because of the difference in cognitive processes involved in responding to an item visually as opposed to aurally.

Substantial modifications occur when significant changes are made to the original assessment, such as changes to the wording or response options. Coons et al [7] suggest that this can fundamentally change the properties of the original instrument and the migrated instrument should be treated as a brand new instrument requiring full psychometric testing.

Prior to the Coons et al.’s [7] framework being established, Gwaltney et al. [8] performed a meta-analysis of equivalence studies (excluding those conducted with IVRS) that had been conducted up until 2006, including studies directly assessing the equivalence of paper and ‘computer’ versions of PROMs used in clinical trials. As this meta-analysis was conducted before Coons et al.’s [7] recommendations were published, the rationale provided for conducting equivalence testing is broad. The approach that Gwaltney et al. [8] supported, and thus the basis of conducting their meta-analysis, was to provide evidence on quantitative equivalence between modes of administration.

The present study was conducted to provide further evidence on the equivalence between questionnaire scores obtained from paper administration and after migration onto one or more electronic platforms. In order to provide this evidence, a systematic review and meta-analysis was performed on equivalence studies conducted since 2007, i.e., since the conduct of Gwaltney et al’s [8] meta-analysis. It was expected that as a consequence of recent advances in technology, the electronic platforms to which the questionnaires are migrated, such as tablets, laptops and smart phones, will be more variable, but that they will be easier to use and will not require prior experience. Ease of use of electronic devices has been shown to result in better compliance and satisfaction [9], therefore reducing potential bias even if respondents are less technologically competent. Thus we hypothesised that the meta-analysis would again show high equivalence scores for instruments migrated to a different administration mode.

Studies that had migrated a questionnaire to an IVRS were also included in the present study; these studies had been excluded from Gwaltney et al’s 2008 analysis [8]. IVRS is frequently used in clinical research [10] and it is considered to be a more substantial change to migrate from paper to IVRS than, for example, to a tablet or smart phone [7], and so we sought to explore the equivalence of scores obtained using this platform.

The present study also explores potential publication bias in the literature. It is possible that studies which demonstrate a lack of equivalence are not submitted for publication, thus risking giving a false impression of the success of migration to and between electronic platforms.

## Methods

### Searching

In order to conduct a refined search in this area of literature, the papers that were included in the Gwaltney et al. [8] review were searched for the indexed terms used in three databases: Embase, Medline and PsycInfo. From this list of indexed terms, those terms that were appropriate to re-running the search were highlighted (e.g., questionnaires, microcomputers, mobile devices, crossover design). A list of terms was created under three headings: ‘PROMs’ , ‘equivalence’ and ‘technology’. Using appropriate Boolean operators, these terms were used in separate searches run in the three databases, with limits placed on the last 6 years (Jan 2007 - Dec 2013) and selecting human studies only.

Once the three searches had been run, the results were exported to Reference Manager to amalgamate the abstracts. The search was further refined by searching through the first 100 abstracts to identify any other relevant indexed terms. This refinement was conducted so that current terminology, which may not have shown up in Gwaltney et al. [8], could be used in the new search. After identifying additional search terms, the final search terms were produced and the searches rerun in the three databases. This search yielded 2,271 abstracts. Additional grey literature was examined by searching conference proceedings of relevant conferences (ISPOR and ISOQOL), the clinical trials registry, and by searching secondary references of articles included in the main search. A further 318 records were identified using this approach.

### Inclusion criteria

A number of criteria were specified to select appropriate studies for inclusion in the review and subsequent analysis. To be included, abstracts and full-text papers/posters had to describe a study which (a) was based on the numeric equivalence of questionnaire scores and no other types of equivalence such as conceptual equivalence, (b) include two different modes of administration, (c) administer a PROM, and (d) provide a statistical result of the equivalence of two questionnaires’ scores (e.g., intra-class correlation coefficient (ICC), Pearson’s correlation, mean difference). The abstract review was conducted by one researcher, who then conferred with another researcher regarding the exclusion of an abstract. Full-text papers/posters were sought for abstracts meeting the criteria. If the abstract suggested that the study might be suitable, but it did not provide details of any of these four criteria, the full-text paper/poster was also sought to assess the study based on these same criteria and to decide whether or not the study should be included. Each full-text paper/poster was then reviewed once by the first researcher, and then a second time by the other researcher, to determine whether or not the study met the inclusion criteria.

**Fig. 1:**
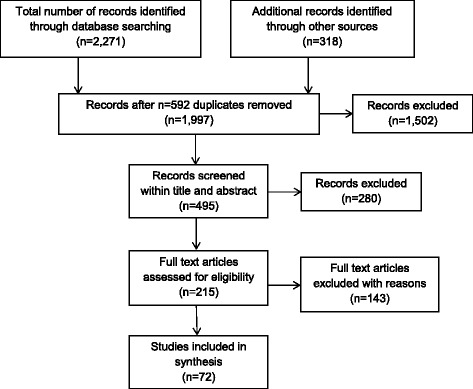
Flow chart showing process of identification and selection of studies for synthesis

The total number of records identified using each of the database and grey literature approaches are shown in the study PRISMA diagram Fig. 1, along with the number of duplicates removed (*n* = 592), number of articles removed after title only analysis (*n* = 1502), the numbers of abstracts screened (*n* = 495) and, of these, the number excluded for one or more of the above reasons (*n* = 280); the number of full text papers assessed (*n* = 215) and, of these, the number excluded (*n* = 143) again for one or more of the above reasons; and the total number of studies meeting the criteria and included in the synthesis (*n* = 72).

### Data analysis

#### Data extraction

For all 72 studies that were included in the meta-analysis, the following data were extracted: (a) name and details of PROM, (b) disease area, (c) study design (parallel groups design or cross-over design), (d) the modes of administration used and details of how these were implemented, (e) mean age and standard deviation (SD) of the participants, (f) the statistic used and the result, and (g) the administration interval. Key features of each study were also identified using a modified data extraction proforma guided by the Strengthening the Reporting of Observational Studies in Epidemiology (STROBE) statement [11]. This data extraction process also served as a critical appraisal process of each study but was not used to exclude any studies from the analysis.

The mean (SD) age of participants was extracted where it was presented. If it was not, then the median age was extracted, or the mean age was calculated from either the presented frequency distribution (with SD also calculated) or the average of minimum and maximum age. Data on the equivalence between the administration modes was extracted for measures of correlation and mean difference. The data on the correlation between questionnaire administrations was extracted as an ICC, Pearson or Spearman correlation coefficient, or a Kappa statistic (weighted or unweighted). Data on mean differences were extracted as a mean difference between administrations with either the presented instrument score standard deviation (SD), standard error (SE), or a 95 % confidence interval (CI), or as separate mean scores for each administration with their own SD, SE, or 95 % CI. The study-specific SD was calculated, where this was not provided, using the sample size and either the SE or 95 % CI. Since it was the magnitude of the difference between administration modes, rather than its direction that is of primary interest, the absolute difference was used in the analysis. This approach also is conservative since it does not allow for positive and negative differences cancelling each other out [8]. Where paired data were available these were used in preference to data from the separate administration groups. Each mean difference was standardized by its extracted SD, meaning a standardized mean difference of 0.5 is a mean difference equivalent to 0.5 (half) of a standard deviation. In addition, since not all studies provided data from which the SD could be calculated, the response scale of each instrument was also extracted (e.g., an instrument scored 0 to 10 has a response scale of 11) and each mean difference standardised by the response scale. Thus, if the mean difference was 2 points on a 100-scale instrument, the standardised mean difference was scored as 2.0 %. This allowed the differences to be measured in terms of scale point difference where information on SD was not available. This was the approach used to compare mean differences by Gwaltney et al. [8].

#### Data synthesis and meta-analysis

Syntheses were conducted first over all individual measures of correlation and all mean differences calculated within each study (i.e., including multiple measures of agreement per study where these were available, such as for different scales within one instrument and different instruments). The main analyses, however, used only one (average) measure of agreement for each study: the average ICC alone; the average ICC, correlation and/or kappa coefficient in each study where multiple coefficients were presented; and the average scaled mean difference. This ensured that no one study made a disproportionate contribution to the analysis. For all analyses, however, syntheses were achieved using a weighted linear combination of the study estimates so as to give more weight to studies of larger size.

The correlation and standardised mean difference data were synthesized using both the ‘metan’ command within Stata v12.1 [12] and Comprehensive Meta Analysis (CMA) v2 software [13] which allows multiple types of data (e.g., mean differences) to be synthesised within the same analysis. Fisher’s z transformations were applied to the correlations within both Stata and CMA. Standard meta-analytic techniques, however, could not be used for the scale-standardised mean differences, as for these no estimate of SD is provided. Instead, simple means and SDs across individual scale-standardised values were calculated to estimate the average scale-point standardised difference. These estimates were calculated over all individual standardised values and over average standardised values calculated for each study.

The degree of heterogeneity between the study estimates was calculated using the I^2^ statistic [14], a measure describing the percentage of total variation across the studies that can be explained by heterogeneity rather than chance. Values of I^2^ lie between 0 % and 100 % with the larger the value the greater the heterogeneity; values of 25 %, 50 %, and 75 % have been proposed to indicate low, moderate, and high heterogeneity, respectively [15]. If values of I^2^ > 0.75 were identified, random effects models were used to synthesise the individual study estimates; fixed effect models were used otherwise (and to explore the effect of any potential moderating factors). Any potentially outlying studies were identified (those with an effect size more than 3.0 SDs away from the pooled effect) and the I^2^ values and pooled effect size recalculated. In exploring the effect of potential moderating variables, fixed effects models were used, with the potential moderating variable treated as a fixed effect. Potential moderating variables considered were, where appropriate: mode of administration/platform (paper vs PC, paper vs PDA, paper vs tablet, paper vs IVRS, PC vs IVRS, tablet vs PC); year of publication (2007, 2008–2010, 2011, 2012–2013; 2007–2010 vs 2011–2013); study design (two variables: randomised cross-over, non-randomised cross-over, within-patient study (a study not formally comparing administration/platform but in which some patients completed more than one mode), parallel groups (for which only analysis of mean differences was possible); non-randomised vs randomised); time interval between administrations (<1 day, 1–5 days, 6–14 days, 15+ days; <1 day, 1–9 days, 10+ days; <1 day, 1+ days), mean age of participants (<28, 28–46, 47–55, 56+ years), sample size (≤50, 51–100, >100 participants) and publication type (abstract/poster vs full-text paper). The modifying effect of these study characteristics on mean score differences and correlations was explored by calculation of pooled values for studies grouped by these factors (year of publication, study design, mode of administration/platform, time interval between administrations, mean age, sample size and publication type). Analyses of variance, with calculation of Q_W_ and Q_B_ statistics [15], where appropriate, were used to compare estimates between groups of studies.

The likelihood of publication bias was estimated with the use of funnel plots along with Duval and Tweedie’s Trim and Fill to estimate the likely number of missing studies (under both fixed and random effects models) and provide estimates of the overall effect after including any identified missing studies. Orwin’s fail-safe N was also used, as in Gwaltney et al. [8], to estimate the number of studies required to bring the observed correlation below 0.75, taking the average correlation as the lowest observed individual study correlation.

## Results

### Study characteristics

Characteristics of all 72 studies meeting the inclusion criteria and included in the meta-analysis are listed in Table 1. Data for four of these studies were available from conference posters and five from abstracts; the remainder from full-text publications. The number of PRO instruments assessed within each study ranged from one to ten, with the number of individual analyses within each study ranging from one to 60. These instruments included generic measures such as the Short Form 36 Health Survey (SF-36) and condition specific measures such as the Rhino-conjunctivitis Quality of Life Questionnaire (RQLQ); for a full list of the instruments included see Table 1. Studies were conducted in over 23 different population types, with the most frequent population being mental health (*n* = 15 studies). The studies included data collected from four different electronic platforms [PC, handheld (PDA/smartphone), IVRS, tablet/touch screen], the most commonly used platform being PC (used in *n* = 43 studies), followed by PDA (*n* = 14 studies), tablet/touch screen (*n* = 8 studies) and IVRS (*n* = 7 studies). The average age of the participants in the studies ranged from 9.58 to 68.3 years, with an overall mean age of 42.9 (SD 17.1) years.

**Table 1:** Characteristics of studies included in the meta–analysis

	Year	Study description									Equivalence indices				Rigour	
Authors and study ID		eMode	D	R	AP	N	Population	Mean age (SD)	eMode comparison	Measure(s)	SMD		Corr	K	Time lag	PC/95 % CIs
Araujo et al. [16]	2012	Web/PC	C	Y	N	21	Asthma	29(10)	Paper–web/PC	ACQ, AQLQ	0.025	–	–	–	4 weeks	PC ✓, CI ✓
Ashley et al. [17]	2012	Web/PC	C	Y	N	111	Cancer	57(13.2)	Paper–web/PC	SDI–21	0.006	0.89	–	–	19 days	Unknown
Basnov et al. [18]	2009	Web/PC	C	Y	N	41	Cancer	47.2(9)	Paper–web/PC	SF–36	–	0.77	–	–	≈2 weeks	CI ✓
Beaumont et al. [19]	2011	Web/PC	P	Y	N	1006	COPD	55(11.4)	Paper–web/PC	COPD–PS	0.012	0.82	–	–	5–7 days	PC ✓, CI ✓
Bennett et al. [20]	2013	Web/PC	C*	Y	Y–A	170	Cancer	56(11)	Paper–web/PC	BFI, NRS LASA QOL	–	0.97	–	–	Next day	Unknown
		IVRS							Paper–IVRS			0.89				
									Web/PC–IVRS			0.88				
Bernabe–Ortiz et al. [21]	2008	PDA	W	N	N	200	Sexual Health	22.9(3.4)	Paper–PDA	[STD Symptoms]	–	–	–	0.86	Immediate	No
Bernstein et al. [22]	2013	Web/PC	W	N	N	116	Sexual Health		Paper–web/PC	SHIM	0.002	0.87	–	–	1 week	Unknown
Bishop et al. [23]	2010	Web/PC	C	Y	N	167	Back Pain	46.28	Paper–web/PC	RMDQ	0.001	0.97	–	–	Immediate	PC ✓
Bushnell et al. [24]	2012	Web/PC	C	Y	Y–P	314	General	53(12.5)	Paper–web/PC	ENSEMBLE MDS	–	0.87	–	–	24 hours	Unknown
Bushnell et al. [25] (a)	2013	Web/PC	C	Y	Y–P	228	General	44.3(13.5)	Paper–web/PC	PDHCO	–	0.90	–	–	1 week	Unknown
Bushnell et al. [26] (b)	2013	Web/PC	C	Y	N	63	Dermatology	50.2(13.6)	Paper–web/PC	PSI	0.023	0.96	–	–	24 hours	Unknown
Carlbring et al. [27]	2007	Web/PC	C	Y	N	494	Panic disorder	37.6(10.9)	Paper–web/PC	BSQ, ACQ, MI, BAI,	0.021	–	0.90	–	<36 hours	PC ✓
										BDI, QOLI, MADRAS						
Chen et al. [28]	2007	Web/PC	C	Y	N	150	General	30.8	Paper–web/PC	SF–36	0.013	–	–	–	10 minutes	PC ✓, CI ✓
Clayer et al. [29]	2011	Web/PC	W	N	N	46	Cancer	53.5(13.9)	Paper–web/PC	TESS	0.005	0.97	–	–	7 days	CI ✓
Coles et al. [30]	2007	Web/PC	C	Y	N	105	OCD	19.01(1.41)	Paper–web/PC	OCI, OBQ–44	0.027	–	0.78	–	≈2 days	No
Cook et al. [31]	2007	Tablet	C	Y	N	80	Depression	44.1(11.6)	Paper–Tablet	QIDS–SR16	0.007	0.99	–	–	Immediate	CI ✓
Cubo et al. [32]	2012	Web/PC	C	Y	N	42	Parkinson’s	64.7(9)	Paper–web/PC	PDQ–39, NMSQ, UPDRS	–	0.82	–	–	<5 days	PC ✓, CI ✓
										II, UPDRS IV						
Dalal et al. [33]	2011	Web/PC IVRS	C	Y	N	149	COPD	53.1(10)	Paper–web/PC	LFQ	0.014	0.81	–	–	1 week	CI ✓
									Paper–IVRS		0.005	0.93				
Dunn et al. [34]	2007	IVRS	W	N	N	99	Sexual function	31	Paper–IVRS	CSFQ	–	–	0.91	–	Unknown	Unknown
Dupont et al. [35]	2009	Tablet	W	Y	N	56	Cancer	54(13)	Paper–Tablet	FACT–G (Social Well–Being Subscale)	0.035	–	–	–	1 minute	PC ✓, CI ✓
Gibbons et al. [36]	2011	PDA	C	Y	N	12	Appetite	25.6(6.3)	Paper–PDA	VAS	–	–	0.93	–	30 minutes	CI ✓
Godfrey et al. [37]	2013	Web/PC	C	Y	N	35	Shoulder injury	48	Paper–web/PC	WORC	0.016	0.89	–	–	Immediate	PC ✓
Griffiths–Jones et al. [38]	2012	Web/PC	C	Y	Y–A	47	Hip injury		Paper–web/PC	Oxford hip score,	0.004	0.97	–	–	1 week	PC ✓, CI ✓
										McCarthy hip score, UCLA activity, howRu						
Gudbergsen et al. [39]	2011	Tablet	C	Y	N	20	Osteoarthritis	66.5(7)	Paper–Tablet	KOOS, VAS (pain,	0.024	0.95	–	–	5 minutes	PC ✓, CI ✓
										function, global), SF– 36, PainDirect						
Handa et al. [40]	2008	Web/PC	C	Y	N	43	Gynecology	52(13)	Paper–web/PC	PFDI–20, PFIQ–7	–	0.86	–	–	<6 weeks	PC ✓, CI ✓
Heiberg et al. [41]	2007	PDA	C	N	N	38	Rheumatology	58.4(12.9)	Paper–PDA	VAS, SF–36, m–HAQ	0.010	–	–	–	3 weeks	Unknown
Hollandare et al. [42]	2010	Web/PC	C	Y	N	87	Depression	41.1(13)	Paper–web/PC	MADRS–S, BDI–II	0.012	–	0.87	–	≈10 days	No
Hollen et al. [43]	2013	PDA	W	N	N	86	Cancer	67	Paper–PDA	LCSS	–	0.92	–	–	15 minutes	CI ✓
Inman et al. [44]	2012	Web/PC	W	N	Y–A	1439	Rheumatology	56.5(14)	Paper–web/PC	HAQ–II, Pain, GA,	0.006	–	–	–	6 months	Unknown
										Fatigue						
Jaspan et al. [45]	2007	PDA	C	Y	N	212	Sexual beh.	14.5(2.75)	Paper–PDA	Individual items	0.035	–	0.72	–	2 weeks	CI ✓
Jones et al. [46]	2008	Web/PC	P	N	N	183	Mental health	20.1(2.6)	Paper–web/PC	PIQ, LSHS–R	0.016	–	–	–	N/A	No
Juniper et al. [47]	2007	PDA	C	Y	N	70	Rhino–conjunctivitis	41	Paper–PDA	RQLQ	0.014	0.90	–	–	2 hours	No
Juniper et al. [48]	2009	PDA	C	Y	N	68, 27	Asthma & Rhino– conjunctivitis	41	Paper–PDA	AQLQ(S), ACQ, RQLQ(S)	–	0.89	0.89	–	2 hours	CI ✓
Junker et al. [49]	2008	PDA	C	Y	N	200	Chronic pain	57	Paper–PDA	Average, present, worst pain & PainDetect	0.026	–	–	–	<1 day	PC ✓, CI ✓
Kajander et al. [50]	2007	PDA	W	N	Y–A	15	IBS	42	Paper–PDA	IBS	0.008	0.96	–	–	Unknown	CI ✓
Koolen et al. [51]	2011	Web/PC	C*	Y	N	156	Asthma	11.25(1.9)	Paper–web/PC	C–ACT, ACT	–	0.83	–	–	<5 days	PC ✓, CI ✓
Lam et al. [52]	2009	IVRS	C	Y	N	64	IBD	43.1(13.8)	Paper–IVRS	SIBDQ	0.013	–	0.89	–	≈7 days	No
Lee et al. [53]	2009	Tablet	C	Y	N	261	Asthma	40.8(12.1)	Paper–Tablet	A–QOL	–	–	–	0.85	Unknown	Unknown
Luce et al. [54]	2007	Web/PC	C*	Y	N	74	Eating disorders	15.4(0.3)	Paper–web/PC	risk for eating disorders	0.025	–	–	0.74	1 week	Unknown
Lundy and Coons [55]	2011	IVRS	C	Y	N	113	General	61.5	Paper–IVRS	EQ–5D index & VAS	0.018	0.89	–	0.71	3 days	Unknown
Lundy et al. [56]	2013	IVRS	C	Y	N	139	Cancer	61.5	Paper–IVRS	QLQ–C30	0.015	0.82	–	–	2 days	PC ✓, CI ✓
Mackenzie et al. [57]	2011	Web/PC	C	Y	N	56–63	Psoriatic	53	Paper–web/PC	HAQ, SF–36, mFSS,	0.003	0.95	–	–	Consecutive	CI ✓
							Arthritis			FACIT–F, DLQI, BASDAI, BASFI, BASG, BASQoL, EQ–5D						
Marceau et al. [58]	2007	PDA	C	Y	N	36	Chronic Pain	48 (8)	Paper–PDA	Pain diary	0.020				2 weeks	No
Matthew et al. [59]	2007	PDA	C*	Y	N	39–53	Cancer	67.2(10.3)	Paper–PDA	IPSS	0.042	0.85			30 minutes	No
McCarrier et al. (a) [60]	2011	Web/PC	C*	Y	Y–A	258	Mental Health	48.6(13.5)	Paper–web/PC	PHQ– 4	–	0.86	–	–	1 week	Unknown
McCarrier et al. (b) [61]	2011	Web/PC	C*	Y	Y–P	256	General	48.6(13.5)	Paper–web/PC	MOS–SSS	–	0.89	–	–	1 week	Unknown
McCarrier et al. [62]	2013	Web/PC	C*	Y	Y–P	230	Multiple	44.3(13.5)	Paper–web/PC	DBS	0.020	0.88	–	–	24 hours	PC ✓, CI ✓
Mundt et al. [63]	2010	IVRS	W	N	N	62	Mental health		Paper–IVRS	DAS–A	0.019	0.87	0.89	–	24 hours	Unknown
Parnell et al. [64]	2011	Web/PC	C	Y	N	50	Pelvic floor	50.4(11.6)	Paper–web/PC	PISQ–12	0.008	0.88	–	–	2 weeks	PC ✓, CI ✓
Raat et al. (a) [65]	2007	Web/PC	P	Y	N	933	Child health	14.7(0.68)	Paper–web/PC	CHQ	0.010	–	–	–	–	Unknown
Raat et al. (b) [66]	2007	Web/PC	P	Y	N	933	Asthma	14.7(0.68)	Paper–web/PC	ISAAC	0.008	–	–	–	N/A	PC ✓
Ramachandran et al. [67]	2008	Tablet	C	Y	N	314	General	35.5(14)	Paper–Tablet	EQ VAS	0.014	0.75	–	–	Unknown	Unknown
Read et al. [68]	2009	Web/PC	C	N	N	38	Trauma	19.6(1.5)	Paper–web/PC	TLEQ, PCL–C	0.058	–	0.69	–	1 week	No
Richardson et al. [69]	2009	Web/PC	P	N	N	354	Smoking Dx	16(1.55)	Paper–web/PC	Social and emotional dependence, physical and sensory	0.052	–	–	–	N/A	No
Richter et al. [70]	2008	Tablet	C	Y	N	153	Rheumatology	45.7(14.4)	Paper–Tablet	FFbH. HAQ. BASDAI, SF–36	0.001	–	0.97	–	Unknown	Unknown
Salaffi et al. [71]	2009	Tablet	C	Y	N	87	Rheumatology	65	Paper–Tablet	VAS–GH/Pain/PGA, ROAD, TJC	0.008	0.92	–	–	60 minutes	CI ✓
Saunders et al. [72]	2007	Web/PC	C*	N	N	50	Hearing loss	65.6(8.9)	Paper–web/PC	ALHQ	–	–	0.74	–	9–10 days	CI ✓
Shervin et al. [73]	2011	Web/PC	W	N	N	61	Osteoarthritis^a^	63	Paper–web/PC	The Harris hip score,	0.017	–	0.85	–	Immediate	PC ✓
		Tablet					Others		Paper–Tablet	WOMAC, SF–36, EQ–	0.005		0.84			
									Tablet–web/PC	5D, UCLA activity score	0.013		0.90			
Swartz et al. [74]	2007	PDA	C	Y	N	756	Mental Health	55(13)	Paper–PDA	CES–D	0.023	–	–	–	Immediate	Unknown
Thoren et al. [75]	2012	Web/PC	C	Y	N	53	Hearing loss	68.3(11.3)	Paper–web/PC	HHIE, IOI–HA, SADL, HADS	0.016	–	0.73	–	3 weeks	No
Tiplady et al. [76]	2010	PDA	C	Y	N	43	Rheumatoid Arthritis	57	Paper–PDA	HAQ–DI, EQ–5D, BPI, MPQ–SF, FACIT–F, SF–36, SARA	0.009	0.88	–	–	1 hour	CI ✓
Turvey et al. [77]	2012	IVRS	W	N	N	51	Mental Health	68(8)	Paper–IVRS	PHQ–9	0.036	0.65	–	–	1 week	No
Vallejo et al. [78]	2007	Web/PC	W	N	N	185	Mental Health	27.4(10.0)	Paper–web/PC	GHQ–28, SCL–90–R	0.020	–	0.69	–	≈17 days	No
Vallejo et al. [79]	2008	Web/PC	C	Y	N	40	Mental Health	22.2	Paper–web/PC	GHQ–28, SCL–90 (Spanish)	–	–	0.84	–	<1 week	No
Varni et al. [80]	2008	Web/PC	C	Y	N	92	Diabetes	13.2(3.42)	Paper–web/PC	PedsQL 4.0	0.007	0.89			<5 minutes	No
Vinney et al. [81]	2011	PDA	C	Y	N	19	Speech	9.58(1.22)	Paper–PDA	PedsQL 4.0.	0.008	0.86	–	–	3 weeks	PC ✓
Whitehead et al. [82]	2011	Web/PC	P	Y	N	1034	Mental Health	24.07(8.5)	Paper–web/PC	HADS, SF–36v2, FSI and Fatigue item	0.012	–	–	–	N/A	CI ✓
Wijndaele et al. [83]	2007	Web/PC	W	N	N	130	Mental health	46.5	Paper–web/PC	GHQ–12, SCL–90–R, MOS–SSS, PSS, UCL	–	0.76	–	–	1 week	No
Wu et al. [84]	2009	Web/PC	C	Y	N	34	Heart failure	49(14.2)	Paper–web/PC	KCCQ, MLHFQ, SCHFI	0.027	–	–	–	2 weeks	CI ✓
Young et al. [85]	2009	Web/PC	C	Y	N	69	Child health	11(1.55)	Paper–web/PC	ASK, PedsQL	0.013	0.81	–	–	2 weeks	CI ✓
Yu and Yu, 2007 [86]	2007	Web/PC	P	Y	N	1171	Mental health		Paper–web/PC	CES–D Chinese	0.019	–	–	–	N/A	No
Zimmerman & Martinez [87]	2012	Web/PC	W	N	N	53	Mental health	45.1(12.3)	Paper–web/PC	CUDOS	0.009	0.96	–	–	<2 days	No

*C* crossover, C* 3/4–group crossover, *P* parallel, *W* within subjects, *D* design, *R* randomisation, *AP* abstract/poster, *K* kappa (weighted or unweighted), *SMD* scaled mean difference (study average), *Time lag* time between administrations, *PC/95 % CIs* power calculation or precise 95 % confidence intervals, *Dx* diseases

*ACQ* Agoraphobic Cognitions Questionnaire, *ACT* Asthma Control Test, *ALHQ* The Attitudes towards Loss of Hearing Questionnaire, *AQA* Asthma Control Questionnaire, *AQLQ(S)* Asthma Quality of Life Questionnaire, *ASK* Activities Scale for Kids, *A–QOL* Asthma–specific Quality of Life, *BAI* Beck Anxiety Inventory (BAI), *BASDAI* Bath Ankylosing Spondylitis Disease Activity Index, *BASFI* Bath Ankylosing Spondylitis Functional Index, *BASG* Bath Ankylosing Spondylitis Global Score, *BASQoL* Ankylosing Spondylitis Quality of Life Instrument, *BDI* Beck Depression Inventory, *BFI* Bowel Function Instrument, *BPI* Brief Pain Inventory, *BSQ* Body Sensations Questionnaire, *C–AC* Childhood Asthma Control Test, *CES–D* Center for Epidemiologic Studies Depression, *CHQ* Child Health Questionnaire, *COPD* Chronic Obstructive Pulmonary Disease, *COPD–PS* COPD Population Screener, *CSFQ* Changes in Sexual Functioning Questionnaire, *CUDOS* Clinically Useful Depression Outcome Scale, *DAS–A* Assessment Scale for Anxiety, *DBS* 4 item Disease Burden Scale, *DLQI* Dermatology Life Quality Index, *ENSEMBLE MDS* a battery of phenotypic patient–reported instruments administered at baseline in clinical studies, *EQ–5D* EuroQOL–5 Dimensions, *EQ VAS* EuroQOL Visual Analog Scale, *FACT* Functional Assessment of Cancer Therapy, *FACIT–F* The Functional Assessment of Chronic Illness Therapy Fatigue, *FFbH* Hannover Functional Ability Questionnaire, *FSI* Fatigue Symptom Inventory, *GA* Global Assessment, *GHQ–12* General Health Questionnaire–12 items, *GHQ–28* General Health Questionnaire–28 items, *HAQ–II* Health Assessment Questionnaire II, *HAQ–DI* Health Assessment Questionnaire Disability Index, *HHIE* Hearing Handicap Inventory for the Elderly, *howRU* a short generic tool for measuring patient–reported outcomes, *IBD* Irritable Bowel Disease, *IBS* Irritable Bowel Syndrome, *IOI–HA* International Outcome Inventory for Hearing Aids, *IPSS* International Prostate Symptom Score, *ISSAC* Eight items from the International Study of Asthma and Allergies in Childhood, *KCCQ* Kansas City Cardiomyopathy Questionnaire, *KOOS* Knee injury and Osteoarthritis Outcome Score, *LCSS* Lung Cancer Symptom Scale, *LFQ* Lung Function Questionnaire, *LSHS–R* The revised Launay–Slade Hallucination Scale, *MADRAS* Montgomery Asberg Depression Rating Scale, *MADRS–S* Montgomery Asberg Depression Rating Scale, *m–FSS* The modified Fatigue Severity Scale, *m–HAQ* Modified Health Assessment Questionnaire, *MI* Mobility Inventory, *MLHFQ* Minnesota Living with Heart Failure Questionnaire, *MOS–SSS* Medical Outcomes Study Social Support Scale, *MPQ–SF* McGill Pain Questionnaire, *NMSQ* Non–Motor Symptoms Questionnaire; *NRS LASA QoL* Numerical Rating Scale Linear Analogue Self–Assessment of Quality of Life, *OBQ–44* Obsessive Beliefs Questionnaire–44, *OCI* Obsessive Compulsive Inventory, *PCL–C* Posttraumatic Stress Disorder Checklist – Civilian Version, *PDHCO* The Provider–Dependent Health Care Orientation, *PDQ–39* Parkinson´s Disease Questionnaire, *PedsQL 4.0* Pediatric Quality of Life Inventory, *PFDI–20* Pelvic Floor Distress Inventory–20, *PFIQ–7* Pelvic Floor Impact Questionnaire–7, *PHQ–4* Patient Health Questionnaire, *PHQ–9* Patient Health Questionnaire, *PIQ* The Persecutory Ideation Questionnaire, *PISQ–12* Pelvic Organ Prolapse/Urinary Incontinence Sexual Function Questionnaire, *PSI* Psoriasis Symptom Inventory, *PSS* Perceived Stress Scale, *QLQ–C30* Quality of Life Questionnaire – Cancer 30 items, *QOLI* Quality Of Life Inventory, *QUIDS–SR16* 16–item Quick Inventory of Depressive Symptomatology Self–Rated, *RMDQ* Roland Morris Disability Questionnaire, *ROAD* Recent–Onset Arthritis Disability questionnaire, *RQLQ(S)* Rhinoconjunctivitis Quality of Life Questionnaire, *SADL* Satisfaction with Amplification in Daily Life, *SARA* Subjects Assessment of Rheumatoid Arthritis, *SCHFI* Self–Care of Heart Failure Index, *SCL–90–R* Symptoms Check–List–90–Revised, *SDI–21* Social Difficulties Inventory, *SF–36* Short Form Survey 36 items, *SHIM* Sexual Health Inventory for Men, *SIBDQ* The Short Inflammatory Bowel Disease Questionnaire, *TESS* Toronto Extremity Salvage Score, *SS–5* Perceived Social Support, *TESS* The Toronto Extremity Salvage Score, *TIBI* Total Illness Burden Index Truncated Questionnaire, *TJC* Tender Joint Count, *TLEQ* Traumatic Life Events Questionnaire, *UCLA–A* University of California at Los Angeles activity score, *UCLA–A* Utrecht Coping List, *UPDRS II* Unified Parkinson's Disease Rating Scale II, *UPDRS IV* Unified Parkinson's Disease Rating scale IV, *VAS* Visual Analog Scale, *VAS–GH* Visual Analog Scale – General Health, *WOMAC* Western Ontario and McMaster Universities Osteoarthritis Index, *WORC* The Western Ontario Rotator Cuff Index

^a^When reported as ≥0.85 this is recorded as 0.85

### Overall relationship between paper and electronic assessments

#### Mean differences

There were 307 individual estimates of group mean difference (independent group differences or, in preference, paired differences) either with a standard deviation (SD) or with data from which a standard deviation could be calculated. These estimates had low variability with an I^2^ of 33.47; the fixed effects pooled estimate of absolute mean difference was 0.037 (95 % CI 0.031 to 0.042).

There were 355 individual estimates of group mean differences which could be standardised by the scale score. The mean scale-standardised difference was 0.0180 scale points, i.e., 1.80 % of the score range, (range = 0.00 to 0.13, 0 to 13 %; SD = 0.021) with the upper bound of the 95 % CI (0.015 to 0.020) indicating that the difference in absolute scores between platforms is likely to be at most 2.0 %. The mean difference was within 5 % of the scale score in 93 % of estimates. For the scale-standardised scores averaged over 54 studies with data on mean differences, the mean scale-standardised difference was slightly smaller at 0.0167 scale units (range = 0.001 to 0.058; SD = 0.012), with 95 % CI 0.013 to 0.020. Two of these studies [33, 72] had data on different platform comparisons, giving 57 mean differences by study and platform in total (platform-specific comparisons), with a mean of 0.0163 (range 0.001 to 0.057; SD = 0.012), with 95 % CI 0.013 to 0.019, and 97 % having a value within 5 % of the scale score.

#### Correlations

**Fig. 2:**
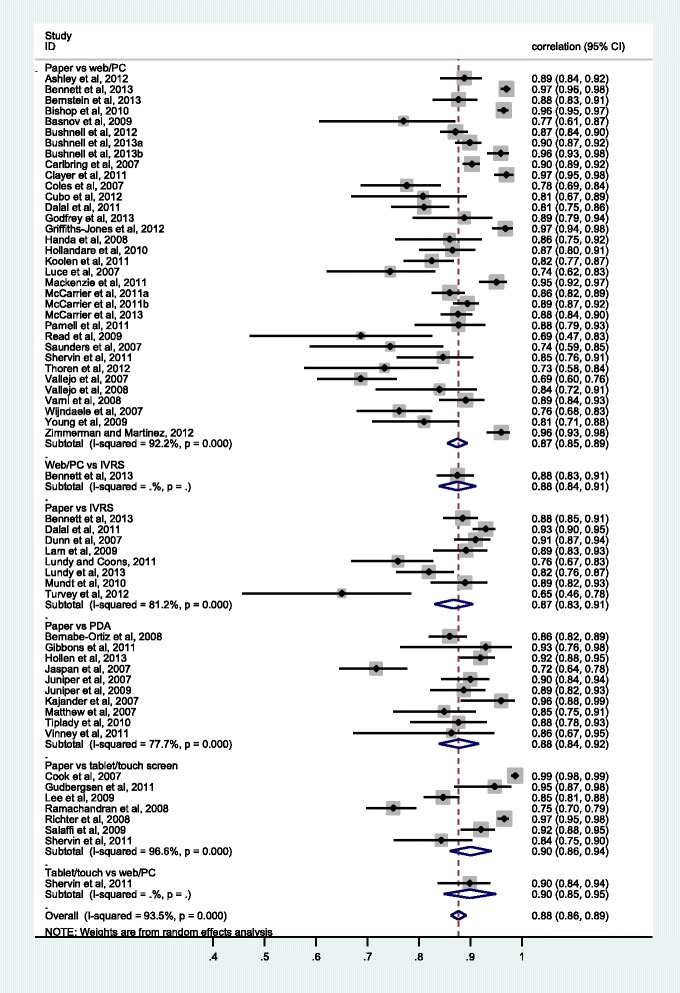
Forest plot of the 61 correlation coefficients averaged over each study and platform

435 individual correlations were extracted from all 72 studies, these being highly variable, with an I^2^ of 93.75 %. The random effects pooled correlation coefficient was 0.875 (95 % CI 0.867 to 0.884). Correlations averaged over the values in each of 56 studies with available data (one study providing values for two different platform comparisons [33] and two studies three different comparisons [20, 72]; i.e., 61 platform-specific values in total) are shown in Fig. 2, grouped by platform comparison. There was a high degree of variability among the studies, with an I^2^ of 93.5. The random effects pooled estimate was 0.884 (95 % CI 0.863 to 0.901). Similarly, average ICCs alone extracted from 39 studies (42 estimates) had an overall random effects pooled estimate of 0.900 (95 % CI 0.878 to 0.918) and an I^2^ of 91.5.

Examination of the standardised residuals for each of the 61 (study and platform specific) estimates, with stepwise exclusion of studies with standardised residuals ≥ |3.0| [full details available from the authors], led to 20 studies being excluded [20a, 23, 26, 27, 29, 30, 31, 33b, 38, 45, 54, 55, 57, 67, 68, 69, 77, 78, 83, 87] with the remaining 41 values having a moderate [15] I^2^ value of 54.39, with a random effects pooled value of 0.874 (95 % CI 0.862 to 0.885) and fixed effects pooled value of 0.875 (95 % CI 0.867 to 0.882).

### Analysis of moderator variables

#### Mean differences

In terms of factors which might explain the observed heterogeneity, for the 307 individual standardised mean differences (data shown in Table 2), while there was no overall difference in the values from studies published in 2011–2013 and those published in 2007–2010, agreement was greater (i.e., pooled standardised mean differences smaller) in the values from studies published in 2008–2010 and 2011, compared with those from studies published in 2007 and 2012–2013 (*p* < 0.001).

**Table 2:** Pooled fixed effects (standardised mean differences) by year of publication, study design, platform, time interval between administrations, age, sample size and publication type for the 307 available standardised mean differences

Study characteristic	*N* = 307	
	N	Mean difference (95 % CI)
Year of publication		
2007	98	0.051 (0.040–0.062)
2008–2010	76	0.022 (0.011–0.033)
2011	69	0.031 (0.020–0.041)
2012–2013	64	0.047 (0.035–0.058)
		*p* < 0.001
2007–2010	174	0.036 (0.028–0.043)
2011–2013	133	0.038 (0.030–0.045)
		*p* = 0.709
Study design		
Randomized cross over	232	0.030 (0.023–0.036)
Non–randomised crossover	12	0.033 (−0.003–0.068)
Within–patient study	40	0.099 (0.077–0.122)
Parallel group study	23	0.046 (0.033–0.060)
		*p* < 0.001
Randomised^a^	256	0.034 (0.028–0.040)
Not randomised	51	0.065 (0.046–0.084)
		*p* = 0.002
Platform		
Paper vs IVRS	40	0.053 (0.038–0.069)
Paper vs PDA	60	0.106 (0.070–0.142)
Paper vs Web/PC	152	0.038 (0.031–0.045)
Paper vs Tablet/touch screen	51	0.020 (0.009–0.031)
Tablet/touch vs Web/PC	4	0.044 (−0.113–0.221)
		*p* < 0.001
Time interval		
0 (<1 day)	159	0.036 (0.028–0.044)
1 (1–5 days)	61	0.034 (0.024–0.044)
2 (6–14 days)	50	0.036 (0.022–0.051)
3 (15+ days)	37	0.051 (0.032–0.069)
		*p* = 0.460
0 (<1 day)	159	0.036 (0.028–0.044)
1 (1–9 days)	85	0.033 (0.025–0.041)
2 (10+ days)	63	0.055 (0.038–0.072)
		*p* = 0.077
Mean age of participants^b^		
<28 years	86	0.064 (0.051–0.077)
28–46.9 years	37	0.019 (0.009–0.029)
47–55.9 years	51	0.028 (0.017–0.039)
56+ years	125	0.049 (0.038–0.061)
		*p* < 0.001
Sample size		
≤56	114	0.071 (0.055–0.087)
57–116	94	0.024 (0.014–0.035)
>116	99	0.036 (0.029–0.043)
		*p* < 0.001
Publication type		
Abstract/poster	3	0.071 (−0.016–0.158)
Full text publication	304	0.037 (0.031–0.042)
		*p* = 0.436

^a^Patients in 1 within–patient study [20] were randomly assigned to complete 2 versions of 1 of 4 instruments

^b^Four studies [7, 23, 48, 71] did not provide information on the age of their participants

Values from studies comparing paper with tablet devices appeared to have the greatest level of agreement (*p* < 0.001).

In terms of study design, agreement was greater in the 256 values from randomised studies compared with the 51 values from non-randomised studies and in cross-over studies compared with within-patient and parallel group studies (*p* < 0.001). Studies with a longer interval between administrations and 56 or fewer participants had lower levels of agreement (*p* = 0.077). In terms of participant age, the 84 values from studies with a mean of <28 years had the lowest agreement, and the 40 values from studies with a mean of 28–46 years the greatest, *p* < 0.001. There was no significant association with publication type (Table 2).

Using the 57 scale-standardised mean differences averaged across each study and platform, mean(SE) differences were significantly lower (i.e., agreement greater) in the 25 studies published from 2011–2013 than in the 32 from 2007–2010 [0.0128(0.008) vs 0.019(0.013), respectively; *p* = 0.045]. There were no other statistically significant differences in terms of study design, platform, time interval, mean age of participants, study size, and publication type, although the mean(SE) differences in the 3 values from non-randomised cross-over studies [0.023(0.030)] and the 7 from parallel group studies [0.0183(0.015)] were non-significantly larger than those from the 35 randomised cross-over studies [0.0155(0.010)] and the 12 within-patient studies [0.016(0.011)], *p* = 0.702. Similarly, the 20 studies with an interval between administrations of <1 day had smaller mean(SE) differences than the 37 with an interval of 1+ days [0.014(0.011) vs 0.017(0.012), respectively; *p* = 0.356]; and the 12 studies with a mean participant age of <28 years had the largest mean(SE) difference, and the 12 with a mean age of 28–46 years the smallest [0.022(0.017) vs 0.012(0.007), respectively; *p* = 0.068].

#### Correlations

Using the 61 correlations averaged across each study and platform (data shown in Table 3), there was a difference in pooled correlation estimates between studies grouped by publication year, with agreement in earlier years, particularly in 2007, being lower (fixed effects *p* < 0.001). The design of the studies was also significantly associated with the degree of correlation, with the highest agreement being observed in randomized studies and the lowest in non-randomised studies (*p* < 0.001). In terms of platform, 8 studies compared a paper with an IVRS measure, 34 a paper with a PC measure, 10 a paper with a PDA measure, and 7 a paper with a tablet/touch screen measure. The paper vs IVRS comparisons had the lowest pooled agreement and the paper vs tablet the highest. In terms of the time period between administrations, agreement decreased as the time interval increased (*p* < 0.001). The age of the participants also had a significant association with agreement, with the youngest participants (those aged <28 years on average) having the lowest agreement but other age groups generally having comparable levels of agreement. While study size had no significant association with agreement, there was a significant association with publication type, with data extracted from 51 full-text publications having lower levels of agreement than data extracted from 10 abstracts/posters (*p* < 0.001). Relationships assessed using all available 435 correlations were similar, although the association with sample size, with smaller studies having greater agreement, was statistically significant (Table 3).

**Table 3:** Pooled fixed effects (correlations) by year of publication, study design, platform, time interval between administrations, age, sample size and publication type for the 435 available correlations and 61 correlations averaged over each study/platform

Study characteristic	*N* = 61		*N* = 435	
	N	Correlation (95 % CI)	N	Correlation (95 % CI)
Year of publication				
2007	12	0.854 (0.839–0.867)	128	0.835 (0.830–0.840)
2008–2010	17	0.879 (0.868–0.890)	98	0.873 (0.869–0.877)
2011	15	0.876 (0.864–0.888)	128	0.891 (0.886–0.896)
2012–2013	17	0.895 (0.886–0.904)	81	0.877 (0.873–0.882)
		*p* < 0.001		*p* < 0.001
2007–2010	29	0.868 (0.859–0.876)	226	0.852 (0.849–0.856)
2011–2013	32	0.888 (0.881–0.895)	209	0.883 (0.880–0.886)
		*p* < 0.001		*p* < 0.001
Study design				
Randomized cross over	44	0.884 (0.878–0.889)	287	0.876 (0.874–0.879)
Non–randomised cross over	3	0.825 (0.775–0.865)	22	0.825 (0.807–0.841)
Within–patient study	14	0.858 (0.842–0.873)	126	0.828 (0.822–0.833)
		*p* < 0.001		*p* < 0.001
Randomised^a^	45	0.884 (0.878–0.889)	293	0.876 (0.874–0.879)
Not randomised	16	0.853 (0.837–0.867)	142	0.826 (0.820–0.831)
		*p* < 0.001		*p* < 0.001
Platform				
Paper vs IVRS	8	0.845 (0.824–0.864)	54	0.844 (0.836–0.850)
Paper vs PDA	10	0.851 (0.830–0.859)	69	0.851 (0.844–0.859)
Paper vs Web/PC	34	0.886 (0.879–0.893)	197	0.863 (0.859–0.866)
Paper vs Tablet/touch screen	7	0.890 (0.876–0.902)	91	0.877 (0.872–0.881)
Web/PC vs IVRS	1	0.880 (0.841–0.910)	2	0.917 (0.898–0.932)
Tablet/touch vs Web/PC	1	0.899 (0.837–0.938)	22	0.917 (0.908–0.926)
		*p* < 0.001		*p* < 0.001
Time interval				
0 (<1 day)	22	0.901 (0.892–0.909)	223	0.890 (0.887–0.893)
1 (1–5 days)	13	0.891 (0.882–0.900)	83	0.877 (0.874–0.881)
2 (6–14 days)	19	0.852 (0.840–0.864)	91	0.813 (0.805–0.819)
3 (15+ days)	7	0.820 (0.791–0.845)	38	0.779 (0.767–0.791)
		*p* < 0.001		*p* < 0.001
0 (<1 day)	22	0.901 (0.892–0.909)	223	0.890 (0.887–0.893)
1 (1–9 days)	26	0.881 (0.873–0.888)	148	0.862 (0.859–0.866)
2 (10+ days)	13	0.819 (0.799–0.837)	64	0.790 (0.781–0.798)
		*p* < 0.001		*p* < 0.001
Mean age of participants^b^				
<28 years	12	0.794 (0.772–0.814)	80	0.789 (0.781–0.796)
28–46.9 years	15	0.896 (0.888–0.904)	98	0.875 (0.871–0.878)
47–55.9 years	12	0.889 (0.878–0.900)	103	0.877 (0.873–0.881)
56+ years	18	0.880 (0.868–0.891)	141	0.886 (0.881–0.891)
		*p* < 0.001		*p* < 0.001
Sample size				
≤56	20	0.881 (0.863–0.896)	126	0.885 (0.878–0.891)
57–116	21	0.881 (0.870–0.891)	184	0.866 (0.862–0.870)
>116	20	0.887 (0.870–0.884)	125	0.861 (0.858–0.864)
		*p* = 0.817		*p* < 0.001
Publication type				
Abstract/poster	10	0.898 (0.889–0.906)	26	0.905 (0.900–0.910)
Full text publication	51	0.870 (0.864–0.877)	409	0.859 (0.856–0.861)
		*p* < 0.001		*p* < 0.001

^a^Patients in 1 within–patient study [20] were randomly assigned to complete 2 versions of 1 of 4 instruments

^b^Four studies [7, 23, 48, 71] did not provide information on the age of their participants

### Assessment of publication bias

**Fig. 3:**
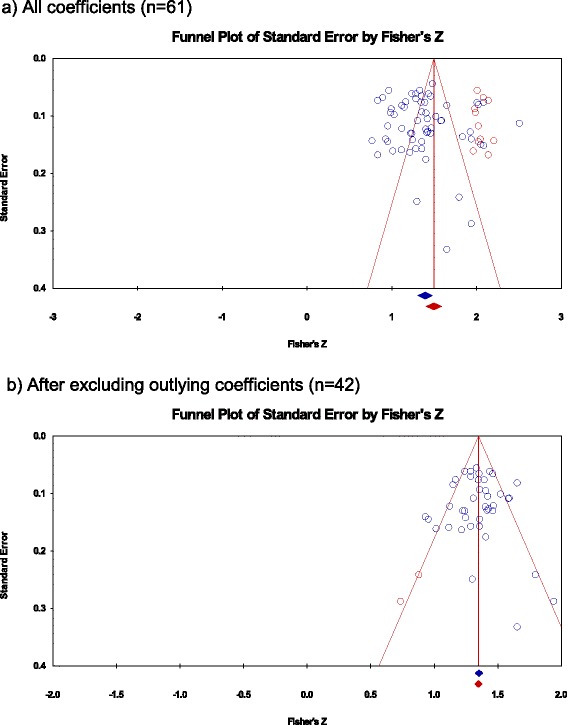
Assessment of publication bias among correlation coefficients averaged over study/platform under a random effects model

Among the total 61 averaged correlations, there was generally little evidence of publication bias (Egger’s regression intercept = 0.886, SE = 1.220, *p* = 0.235; Kendall’s Tau b = 0.070, *p* = 0.211), with no studies estimated as missing using Duval and Tweedie’s trim and fill test under a fixed effects model. Under a random effects model, however, 11 studies were identified as potentially missing to the right of the mean (i.e., studies with a greater degree of agreement; Fig. 3a), their inclusion raising the random effects pooled correlation to 0.904 (95 % CI 0.886 to 0.920). The results were similar after excluding the 10 studies in abstract/poster form: there was little evidence of publication bias among the 51 full text publications (Egger’s regression intercept = 1.006, SE = 1.375, *p* = 0.468; Kendall’s Tau b = 0.061, *p* = 0.521) but with 10 studies (the same number as those excluded) identified as potentially missing to the right of the mean, their inclusion raising the random effects pooled correlation to 0.899 (95 % CI 0.894 to 0.904).

After excluding the 20 outlying valuesthere was generally little evidence of publication bias (Egger’s regression intercept = 0.061, SE = 0.626, *p* = 0.462; Kendall’s Tau b = −0.011, *p* = 0.460), with one study estimated as missing using Duval and Tweedie’s trim and fill test under a fixed effects model and two under a random effects model, both to the left of the mean (i.e., studies with a lesser degree of agreement; Fig. 3b). After including these two studies, the random effects pooled correlation coefficient reduced slightly from 0.874 to 0.872 (95 % CI 0.860 to 0.884) and the fixed effects pooled correlation coefficient from 0.875 to 0.874 (95 % CI 0.866 to 0.881).

Using an average correlation of 0.65 for potentially missing studies, this being the lowest ICC extracted [77], Orwin’s fail-safe N test estimated that 123 missing studies additional to the 61 (79 for the 42 estimates after excluding the outliers) would be needed to bring the observed pooled estimate to <0.75.

## Discussion

The results summarised here indicate that electronic and paper PROMs and different modes of electronic administration produce equivalent scores across a wide range of scenarios (medical conditions and platforms), suggesting that electronic measures can generally be assumed to be equivalent to pen and paper measures. In particular, given the generally high level of agreement across all studies included in this review, there is no evidence that equivalence is compromised by the nature of the condition under investigation, even when the information collected is of a sensitive nature, such as of sexual function [34], sexual health [21, 22], sexual behaviour [45], IBS [50] and IBD [52]. Further analyses exploring the role of measurement domain (e.g. physical or mental health) will be reported in another paper. Of particular note is the fact that, based on the ICCs and the numerically small mean score differences, pen-and-paper scores are equivalent to scores obtained from a variety of electronic platforms – IVRS, handheld, PC, and tablet. While equivalence between paper and IVRS measures appears to be slightly lower than with most other forms of electronic measure (pooled correlation coefficient 0.85 vs 0.89 for paper vs tablet; pooled standardised mean difference 0.053 vs 0.020), the data suggest that the likely true agreement (lower 95 % CI) between paper and IVRS measures is at least 0.82 and thus that there is at least good agreement between data obtained from IVRS and pen and paper measures. This is reassuring given that migration from paper to an IVRS is considered to be a moderate change because of the difference in cognitive processes involved in responding to an item aurally as opposed to visually. These results are also consistent with the results from a recent large study (*N* = 923 adult participants) of the effects of method of administration (paper, PDA, PC, IVRS) on the measurement characteristics of items developed in the Patient-Reported Outcomes Measurement Information System (PROMIS) which strongly supported measurement equivalence across all platforms [88].

The observed mean differences in PROM scores between administration types were small. Taking all mean differences as positive differences, the fixed effects pooled standardized mean difference (mean difference standardised by the SD) of the 307 estimates was 0.037 (95 % CI 0.03 to 0.04). These estimates were also of low variability, with an I^2^ of 33.5. In other words, the average mean difference in scores between electronic and paper measures was small at approximately 0.04 SDs. No comparison with earlier data is possible as Gwaltney et al. [8] did not report on standardised mean differences. Standardising the mean differences by the scale range (rather than the score SD), this difference was equivalent to a scale-standardised mean score difference of 1.8 % or, from the upper bound of the 95 % CI, a difference of at most 2 %. This is consistent with, or slightly smaller than, the 2 % mean scale-standardised difference reported by Gwaltney et al. [8]. Similarly, 93 % of all mean differences in this study were within 5 % of the scale score, exactly the same percentage as reported by Gwaltney et al. [8]. The values were similar when study and platform averaged scale-standardised estimates were used: the 57 values had a mean of 0.0163, with 95 % CI 0.013 to 0.019, and 97 % having a value within 5 % of the scale score.

In terms of ICCs and correlation coefficients, agreement was again high, with a pooled ICC over 42 study-specific estimates of 0.90 (95 % CI 0.88 to 0.92), and a pooled correlation coefficient (all measures of correlation) over 61 study-specific estimates of 0.88 (95 % CI 0.86 to 0.90) and of 0.88 (95 % CI 0.0.87 to 0.88) over all 435 individual estimates. These values are consistent with the pooled summary correlation of 0.90 (95 % CI 0.87 to 0.92) reported by Gwaltney et al. [8], an estimate which was the same irrespective of the specific measure of correlation. There is thus little evidence from both the present study and the earlier one [8] that the measure of correlation used has any influence on the degree of equivalence obtained. This is reassuring given the number of studies not employing the ICC in their assessment of equivalence. The ICC is the statistically correct measure of equivalence when agreement is assessed within (i.e., intra) measures sharing the same metric (i.e., mean and standard deviation); the Pearson correlation (an interclass correlation) is appropriate only when the measures are of a different class and not sharing the same metric [89]. It is also worth noting that not all studies identified in this review employing the ICC, stated which of the six possible ICCs, as described by Shrout and Fleiss in 1979 [90], was employed: whether the model was one-way or two-way, random or mixed, applying to single or average measures, or measuring consistency or absolute agreement. The value of the ICC obtained will depend on the specific model chosen. A full description of the nature of different ICCs is provided by McGraw and Wong, 1996 [89].

The correlation estimates were highly variable in both the current study and Gwaltney et al. [8], with the I^2^ in the current study being >90 %. After excluding outliers, however, the pooled estimates were essentially unchanged. In terms of factors which might explain the observed heterogeneity, agreement was greater in studies reported most recently (2011–2013 vs 2007–1010), in randomised as opposed to non-randomised studies, in studies with an interval between administrations of <1 day (and, overall, the greater the interval the lesser the agreement), and in studies of larger size. In addition, studies including very young children were associated with lower levels of agreement. While these associations were generally of high statistical significance (*p* < 0.001), they were small in magnitude indicating that these factors have only small, albeit precise, effects; agreement is generally high even in those studies with the lowest agreement. Nevertheless, the patterns observed highlight the importance of appropriate study design when assessing equivalence: randomised studies and those with a shorter interval between administrations were associated with greater equivalence, this effect greatest in studies with an interval of fewer than 10 days between administrations. The lower levels of agreement observed in younger individuals (<28 years) may to some extent reflect this effect: four [45, 54, 68, 78] of the five studies [30, 45, 54, 68, 78] conducted in younger individuals with the lowest level of agreement (ICC < 0.80) had intervals between administrations of one week or more.

The same was true of mean differences: average scale-standardised mean differences were lower (agreement higher) in more recent years (2011–2013) compared with earlier years (2007–2010), and randomised studies were associated with greater agreement than non-randomised studies, with the pooled standardised mean difference being 0.035 (95 % CI 0.030 to 0.041) vs 0.065 (95 % CI 0.046 to 0.084), *p* = 0.003. Other design features associated with agreement were the interval between administrations, with agreement being better (mean difference lower and correlation higher) in studies with an interval of <1 day; and mean age of participants, with agreement being better in studies with participants of mean age between 28 and 55 years. Studies in either younger (some studies having participants of mean age <13) or older participants tended to have lower levels of agreement, this consistent with lower levels of familiarity with EDC platforms in the older age group, and perhaps some unreliability in the responses in general from very young children. By definition, correlation coefficients cannot be obtained from parallel group studies; for the 7 estimates from parallel group studies the scale-standardised mean difference was 1.83 % compared with 1.55 % for the 35 estimates from randomised cross-over designs.

Gwaltney et al. [8] also found substantial heterogeneity in their extracted estimates of equivalence and were unable to explain the variability with analysis of the moderating factors (age and computer familiarity). Nevertheless, in this study only 9 of the studies in this analysis reported a correlation that was less than 0.80. Furthermore, this study found little evidence of publication bias; no studies with correlations less than the pooled mean were identified as missing. The identification of 11 possible missing studies with correlations greater than the pooled mean may simply be a reflection of heterogeneity in the data. Finally, as many as 123 studies with a correlation of <0.75 would need to have been conducted and not published in order for the overall effect to have been <0.75. This figure of 123 was greater than the 95 studies similarly estimated by Gwaltney et al. [8] suggesting that the more recent studies are more robust than those identified in the earlier review. There is thus no reason to believe that heterogeneity, and any possible publication bias, should temper the conclusions drawn from this meta-analysis.

In terms of study design, the general critical appraisal process of each study identified some issues which should be taken into account in future studies. For example, only a small proportion of studies (*n* = 18, 25 %) reported on the use of a power calculation when planning the study size and fewer than half used 95 % CIs (*n* = 29, 40 %) in result reporting. These issues relate to the importance of ensuring that the study is large enough to have sufficient power so that the estimated equivalence effect is estimated with sufficient precision so that possible lack of equivalence can be ruled out (i.e., by the 95 % CI excluding all values indicating measurement non-equivalence). Similarly, while it is encouraging to note that parallel studies assessing measurement equivalence are becoming less frequent (of the 7 parallel group studies, 4 (57 %) were reported in the two years from 2007 to 2008, see Table 1), and while the majority of studies identified (*n* = 51, 70.8 %) were randomised cross-over studies, in which participants completed both versions of the PROM in randomly allocated order, only 8 of these [20, 51, 54, 59–62, 72] undertook the equivalence assessment in the context of a full, or almost full, factorial assessment of instrument equivalence. Such full assessment requires the comparison of scores among four groups of respondent: those completing electronic first and then paper (E-P), those completing paper first and then electronic (P-E), those completing two paper versions (P-P), and those completing two electronic versions (E-E). Such assessment, with appropriate statistical analysis (the formal statistical analysis of these 8 studies generally did not, however, capitalise on the study design) allows the expected variability in scores between measures completed on the same platform on two occasions (i.e., test-retest reliability) to be ‘subtracted’, in the context of an analysis of variance, from the variability observed between measures completed on different platforms. This reflects the fact that it is clearly nonsensical to require a greater degree of measurement equivalence between measures on different platforms than is required between one measure on the same platform in the context of the assessment of test-retest reliability: at best the same degree of equivalence should be required.

Such considerations also raise questions about the inherent expectation of equivalence built into such studies. With the documented strengths of electronic modes of administration over paper [5] one might rightly anticipate a quantitative difference in the data captured on different modes of the same questionnaire due to the simple fact that there is better quality data being captured on the electronic system (e.g., fewer items of missing data, no out of range data). The current approach to equivalence studies seems to demand comparability between superior (electronic) and inferior (paper) modes of data capture which risks undermining the true advantages EDC bring to an actual clinical trial over the necessarily artificial setting of the equivalence study.

## Conclusion

The present study strongly supports the conclusion of Gwaltney et al. [8] that PROM data obtained from electronic platforms are comparable to that obtained from paper administration, as well as providing data on the equivalence of PROMs migrated to an IVRS platform, data not included in the earlier Gwaltney et al. study [8]. The high level of agreement seen in this review as well as in the Gwaltney et al review [8] should be reassuring to investigators, authorities and sponsors using electronic devices to collect PROM data, having implications for the use of electronic measures generally and in clinical trials in particular.

Given the weight of the evidence for the equivalence between paper and electronic versions, we propose that equivalence studies should not be necessary to demonstrate the equivalence of validity of a measure that has been migrated to an electronic platform following best practices [7] with minor changes as defined in the ISPOR Taskforce report [7]. These results also suggest that a migration following best practices [7] to an IVRS may not need an equivalence study. Further research into migration principles and standards for IVRS may be needed to support our findings.

This conclusion stands even when estimates of possible unpublished studies are included in our analysis, highlighting the robust nature of instruments migrated from paper onto electronic platforms. We further recommend that common best practices are established among the vendor community (i.e. via the ePRO consortium) to standardize migration principles (i.e. number of items per screen, scrolling through answer options) as well as to define a standard framework for the conduct and publication of equivalence studies.
